# Body Composition and Anthropometric Measurements in Children and Adolescents with Autism Spectrum Disorder: A Case–Control Study in Lebanon

**DOI:** 10.3390/nu16060847

**Published:** 2024-03-15

**Authors:** Melissa Rouphael, Yonna Sacre, Tania Bitar, Christian R. Andres, Walid Hleihel

**Affiliations:** 1 Department of Biology, Faculty of Arts and Sciences, Holy Spirit University of Kaslik, Jounieh P.O. Box 446, Lebanon; melissa.m.roufael@net.usek.edu.lb (M.R.); walidhleihel@usek.edu.lb (W.H.); 2 UMR Inserm 1253 Ibrain, Université de Tours, 37032 Tours, France; christian.andres@univ-tours.fr; 3 Department of Nutrition and Food Sciences, Faculty of Arts and Sciences, Holy Spirit University of Kaslik, Jounieh P.O. Box 446, Lebanon; yonnasacre@usek.edu.lb

**Keywords:** Autism Spectrum Disorder (ASD), typically developing children, body composition, overweight, obesity, developmental stages

## Abstract

The occurrence of overweight and obesity among individuals with Autism Spectrum Disorder (ASD) has become a worldwide epidemic. However, there is limited research on this topic in the Lebanese population. Therefore, this study aimed to assess the differences in anthropometric measurements and body composition variables among Lebanese children, pre-adolescents, and adolescents diagnosed with ASD in contrast to typically developing peers across various developmental stages. Additionally, it aimed to investigate the prevalence of overweight and obesity within this population. A total of 86 participants with ASD and 86 controls were involved in this case–control study, conducted between June 2022 and June 2023. Anthropometric measurements and body composition variables were assessed, followed by statistical analyses to examine the differences between these two groups. The results revealed a significantly higher prevalence of overweight and obesity among individuals with ASD, particularly evident during childhood and pre-adolescence. Additionally, this group exhibited a higher body fat mass and total body fat percentage compared to controls. However, there were no significant differences observed between the two groups during adolescence. These findings emphasize the significance of monitoring and addressing weight status in individuals with ASD to improve their overall health outcomes. Future research directions could focus on investigating the underlying mechanisms contributing to the heightened prevalence of overweight and obesity in this population, ultimately enhancing their quality of life and well-being.

## 1. Introduction

Autism Spectrum Disorder (ASD) is a lifelong neurodevelopmental condition characterized by difficulties in social communication and interaction, complemented by the manifestation of repetitive and restrictive patterns of behaviors or interests [1]. In recent years, there has been a noticeable rise in reported cases of ASD, particularly with a higher incidence observed among boys compared to girls [2,3]. This increase in prevalence could be attributed to several factors, including advancements in diagnostic criteria, improvement in identification and screening techniques, and heightened awareness among both parents and healthcare professionals [4]. In 2014–2015, the estimated prevalence rate ranged from 49 to 513 children per 10,000 across all regions in Lebanon [5]. This rapid increase highlights the necessity for a deeper comprehension of the pathophysiology of this disorder, as the exact etiology of ASD is not yet clear. However, several studies have shown that it involves genetic, epigenetic, and environmental factors [6,7,8,9].

ASD individuals often experience a range of concurrent symptoms, encompassing anxiety, sleep disruptions, gastrointestinal symptoms, epilepsy, and sensory processing difficulties [10]. These sensory dysfunctions, including either a decreased or increased sensitivity to stimuli like light, sound, taste, smell, texture, and touch, may lead to unhealthy eating habits [11,12,13]. In fact, eating difficulties have been noted in the exploration of ASD [14] and persist among many individuals with ASD [15]. The most frequent eating difficulties seen in children with ASD are food selectivity, disruptive mealtime behaviors, and food refusal [16]. They often manifest in early childhood and persist into adolescence. Children with ASD often exhibit specific dietary preferences, typically favoring foods like meat, fish, eggs, beans, and dairy products, while showing a reluctance towards vegetables, seafood, and fruits [17,18]. In severe cases, some may even limit themselves to consuming only liquids or pureed textures [19]. Therefore, individuals with ASD are more prone to experiencing nutritional inadequacies, which predisposes them to malnutrition [20]. Interestingly, several studies have indicated a notably higher prevalence of obesity and overweight among children with ASD compared to typically developing children [21,22,23]. Overweight and obesity in children are often attributed to dietary factors, such as a high energy intake and the consumption of fast food, sugary beverages, and snacks. These dietary habits are prevalent not only in children with ASD but also in typically developing children [24]. However, when it comes to ASD, weight gain has been associated with distinct factors. Numerous studies have identified potential risk factors that may be particularly relevant to children with ASD. These factors include sleep disturbances, a lack of physical activity, motor impairments, the use of antipsychotic medications, genetic predispositions, and food selectivity [25,26]. Moreover, it is fundamental to emphasize that this weight gain can have significant health implications, potentially increasing the risk of conditions such as heart disease, diabetes, and insulin resistance [27].

Assessing the nutritional status of children and adolescents during their developmental stages is essential for the early detection and correction of potential disorders [28]. Anthropometric methods, such as measuring body weight, height, and circumferences (waist and hips), are commonly employed for this purpose. Additionally, standard anthropometric indicators of body composition, including waist–hip ratio (WHR), waist-to-height ratio (WHtR), and body mass index (BMI), are also utilized [29]. In pediatric practice, assessments of body weight, height, and BMI are made using percentile charts that take into account age and gender. However, BMI, a frequently used measure for categorizing body weight, has limitations since it does not provide insights into the composition of individual body components, such as body fat, muscle mass, and body water content [30]. To enhance the monitoring of body weight, it is advisable to employ bioelectrical impedance analysis (BIA), known for its high accuracy and repeatability [31]. Over the past two decades, bioelectrical impedance analysis (BIA) has experienced rapid growth as a method for assessing human body composition [32]. BIA, a simple, non-invasive, convenient, and practical method, could be an option for assessing body composition parameters in ASD children and adolescents. It employs electrical currents and impedance to evaluate both fluid status and various body composition parameters, such as muscle tissue, adipose tissue, and body water [33]. This is especially crucial for adolescents experiencing puberty, a period distinguished by significant shifts in the endocrine system, rapid physical growth, and the emergence of secondary and tertiary sexual traits. These transformations ultimately influence the development of both the morphological and functional aspects of the body [34].

Although some research has delved into the evaluation of the nutritional status of ASD individuals, there is a lack of studies focusing on their body composition, particularly in Lebanon. However, several studies analyzed the body composition of autistic children and adolescents and indicated a higher incidence of overweight and obesity in this population compared to their peers [21,35,36,37].

Therefore, our objective was to investigate the anthropometric measures and body composition variables obtained using BIA across different developmental stages, assess the prevalence of obesity and overweight in a group of Lebanese children and adolescents diagnosed with ASD, and investigate the associated risk factors.

## 2. Materials and Methods

### 2.1. Study Design, Period, and Setting

This case–control comparative study was conducted in all districts of Lebanon, with a focus on children and adolescents aged between 3 and 18 years old. Data collection began in June 2022 and ended in June 2023.

### 2.2. Study Sample

The study sample comprised 86 Lebanese children and adolescents with ASD and 86 typically developing (TD) controls. ASD and TD participants were recruited from specialized institutions, schools, and NGOs located in all districts of Lebanon. Individuals were considered neurotypical if their medical histories and parent or caregiver reports indicated an absence of behavioral difficulties or any sign of motor and language delays. They were matched with individuals diagnosed with ASD in terms of age, gender, and geographic location. The diagnosis of ASD was established on the criteria specified in the Diagnostic and Statistical Manual of Mental Disorders, Fifth Edition (DSM-5), and confirmed through assessments using the Childhood Autism Rating Scale (CARS). Only individuals whose parents or caregivers provided informed consent were included in this study. The exclusion criteria included the following: children below the age of 3 or above 18, children diagnosed with Down syndrome or fragile X syndrome, the presence of neurological and hormonal disruptions, digestive system diseases, genetic dysfunctions, and the absence of informed consent to participate in the study. Furthermore, severe ASD cases were excluded from this study to ensure the feasibility of conducting body composition assessments.

### 2.3. Recruitment Process

Invitation and project description letters were sent to all educational institution headmasters. Upon receiving their approval, parents were informed of the objectives and methodology of this study.

#### 2.3.1. Sociodemographic Information

Parents or caregivers met with the investigator so the following participant information could be elicited: child’s age, gender, and residence.

#### 2.3.2. Anthropometric Assessment

A trained and qualified healthcare professional conducted morning measurements for each participant with precision. Height was measured to the nearest 0.1 cm using a wall-mounted stadiometer (Numed AHP004, Numed, Beirut, Lebanon), and weight was recorded to the nearest 0.1 kg using a digital platform scale (Amber Body Scale, Numed APAFE002-P4). Participants were barefoot and dressed in lightweight clothing during these evaluations. The body mass index (BMI) was then calculated using Quetelet’s formula ($\frac{Weight\;\left(kg\right)}{{Height}^{2}{(m}^{2})}$) and computed via statistical analysis. Moreover, waist circumference (WC) was assessed by placing a Cescorf^®^ inelastic measuring tape (Cescorf, Porto Alegre, Brazil), calibrated to 1 mm, at the midpoint between the lower rib margin and the iliac crest. The weight-for-age z-score (WAZ), height-for-age z-score (HAZ), and body mass index (BMI)-for-age z-score (BAZ) were computed based on the Center for Disease Control (CDC) growth charts [38] for 2–20 years of age, illustrated in Table 1 [39,40].

#### 2.3.3. Body Composition Assessment

Body composition parameters were evaluated with a Biodynamic 450^®^ version 5.1 analyzer (Biodynamics Corporation, Seattle, WA, USA) administered by a trained healthcare professional. All assessments were consistently conducted in the early hours, between 7:00 a.m. and 10:00 a.m., ensuring accuracy and uniformity in the data collection process. During the procedure, participants were instructed to lie down with their limbs extended parallel to their body and away from the chest. To ensure accurate readings, ECG (Electrocardiogram) electrodes were positioned at specific locations, including the dorsal surface of the right wrist, the third metacarpal bone, the anterior surface of the right ankle (between bony prominences), and the dorsal surface of the third metatarsal bone. Before initiating the measurement, we recorded crucial demographic information, including gender, age, height, and weight. Once these details were input into the machine, the measurement process began, typically taking approximately 1 min to complete. Patients were provided with instructions to follow before BIA tests: (1) overnight fasting: patients were advised to abstain from consuming food or beverages overnight, usually for a minimum of 8 h before the examination. This fasting period helped minimize the potential impact of recent meals on the results; (2) exercise restriction: to prevent any temporary alterations in body fluid balance due to physical activity, patients were instructed to refrain from exercise for 24 h leading up to the examination; (3) bladder voiding: patients were encouraged to empty their bladders before the BIA tests. This step aimed to reduce any variations in body fluid content that could affect the accuracy of the results.

### 2.4. Ethical Considerations

This study adhered to the ethical standards and guidelines outlined in the Declaration of Helsinki of 1964 and its subsequent amendments. The study protocol (EC 90010141) underwent review and approval by the Ethics Committee of the Holy Spirit University of Kaslik. Throughout this study, participants were not subjected to any physical or psychological harm. Furthermore, participants’ credentials were treated confidentially, and the study findings were used strictly for academic purposes.

### 2.5. Statistical Analysis

We used the Statistical Package for Social Sciences 22.0 (SPSS^®^ Inc., Chicago, IL, USA) for statistical analyses. Categorical variables were summarized as frequencies (n) and percentage (%), while continuous variables were presented as mean ± standard deviation. A *p*-value less than 0.05 was considered statistically significant. After conducting tests for data distribution normality, it was determined that variables such as height, weight-for-age z-score (WAZ), and BMI-for-age z-score (BMIZ) were normally distributed. Consequently, comparisons of these variables between the two groups were performed using independent-sample *t*-tests. On the other hand, variables such as weight, weight-for-age z-score cut-points (WAZ cut-points), height-for-age z-score (HAZ), height-for-age z-score cut-points (HAZ cut-points), BMI, BMI-for-age z-score cut-points (BMIZ cut-points), waist circumference (WC), fat mass (FM), fat-free mass (FFM), and percentage of body fat (PBF) exhibited non-normal distributions. As a result, the non-parametric Wilcoxon Mann–Whitney test was utilized for comparing these variables between the two groups. Pearson chi-square analysis was used to explore the correlation between age, gender, geographic location, and the occurrence of obesity in ASD. Furthermore, multinomial linear regression analysis was applied to investigate the relationship between age, gender, geographic location, and the likelihood of being overweight and obese.

## 3. Results

### 3.1. Participants’ Sociodemographic Characteristics

**Figure 1:**
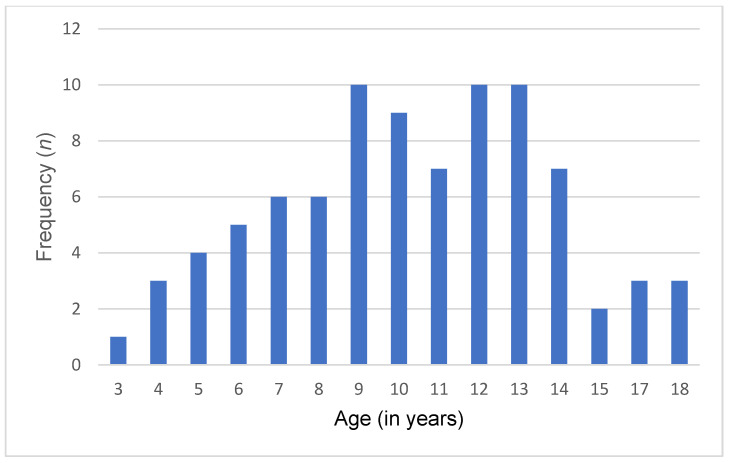
Age distribution in the ASD group.

**Figure 2:**
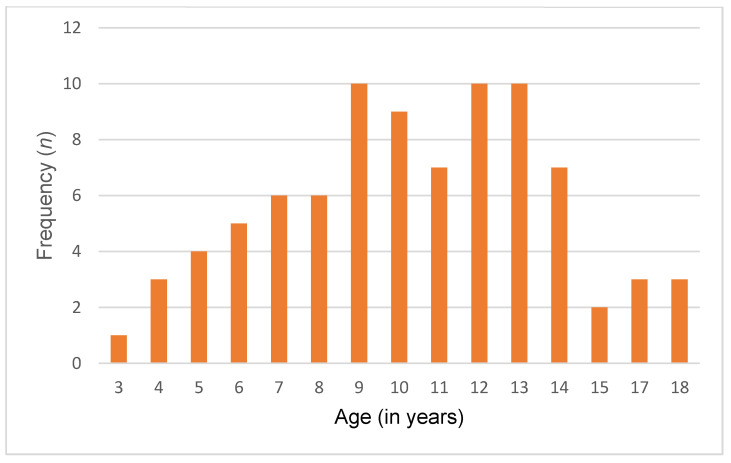
Age distribution in the control group.

A total of 86 Lebanese children and adolescents diagnosed with ASD and 86 typically developing controls were included in this study. The mean age of both groups was 10.4 ± 3.51 years. Among them, 74 (86%) were male, and 12 (14%) were female. Of 86 participants, 35 (40.7%) were young children, 36 (41.9%) were pre-adolescents, and 15 (17.4%) were adolescents. Additionally, participants were recruited from all Lebanese governorates, with the following distribution: Beirut (20.9%), Bekaa (22.1%), North (36.1%), and South (20.9%). Detailed sociodemographic characteristics are presented in Table 2 and Figure 1 and Figure 2.

### 3.2. Anthropometric Assessment

Young children diagnosed with ASD exhibited a notably greater average weight compared to controls (29.7 kg vs. 24.45 kg, *p* < 0.05). This trend persisted into pre-adolescence, with ASD individuals demonstrating significantly higher mean weights (50.88 kg) than controls (39.57 kg). However, during adolescence, no significant difference in mean weight was noted between individuals with ASD (70.44 kg) and typically developing individuals (61.15 kg) (*p*-value = 0.740). Similarly, significant differences were observed in weight-for-age z-scores (WAZ), with higher values in young children and pre-adolescents diagnosed with ASD compared to TD. In adolescence, there was no statistically significant differences between the ASD and control groups. While most participants in both the ASD and TD groups fell within the normal WAZ range, TD controls showed a higher prevalence of low weight across all age groups. Conversely, individuals with ASD exhibited a significantly higher prevalence of obesity, particularly in young children and pre-adolescents, compared to TD controls. However, this prevalence became similar between both groups when transitioning into adolescence. Furthermore, this study found no significant differences in height or height-for-age z-scores (HAZ) between individuals with ASD and TD controls across all age groups. These findings are detailed in Table 3.

**Figure 3:**
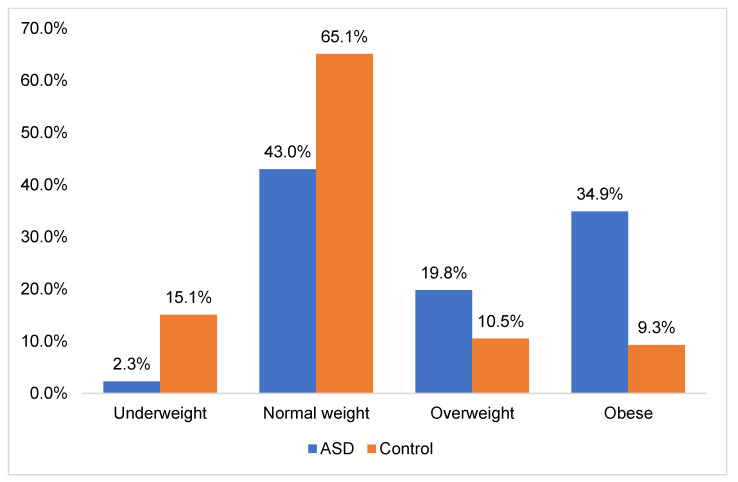
Distribution of obesity status among ASD and TD groups.

Moreover, individuals with ASD exhibited higher BMI and BMI z-score (BMIZ) values compared to TD controls, particularly among young children and pre-adolescents. However, no significant disparities were observed in BMI and BMIZ between individuals with ASD and their matched counterparts during adolescence (*p*-value = 0.389 and *p*-value = 0.355 respectively). In both young children and pre-adolescents diagnosed with ASD, a significant proportion of individuals were classified as either overweight or obese (51% and 58%, respectively) (*p* < 0.05). Conversely, TD controls in these age groups were predominantly categorized as having a healthy weight status. Moreover, the prevalence of overweight and obesity was noted to be higher among adolescents with ASD (53.3%) compared to TD controls (47%), although this difference did not reach statistical significance (*p* > 0.05). These disparities highlight the necessity of monitoring and addressing weight-related issues, especially the increased rates of overweight and obesity in ASD individuals (Figure 3).

Furthermore, the analysis of waist circumference (WC) demonstrated a significantly higher mean WC in young children and pre-adolescents diagnosed with ASD compared to TD controls (67.14 cm and 79.86 cm, respectively) (*p* < 0.05). However, in adolescents, there was no statistically significant difference in mean WC between individuals with ASD (90.2 cm) and TD controls (84.76 cm) (*p* = 0.345). These findings suggest potential disparities in body composition and fat distribution among individuals with ASD during different developmental stages.

### 3.3. Body Composition Assessment

This study uncovered significant differences in body composition among individuals with ASD and their neurotypical counterparts during different developmental stages. Notably, both young children and pre-adolescents with ASD exhibited a notably higher fat mass than the control group (*p* < 0.05). While fat-free mass was also higher in the ASD group compared to the control group across different age groups, these differences were not statistically significant (*p* > 0.05). Furthermore, a more pronounced disparity was observed in total body fat percentage, with ASD-diagnosed young children and pre-adolescents displaying a significantly elevated percentage compared to the control group (*p* < 0.05). Conversely, when analyzing adolescents, the differences in FM, FFM, and BPF between the ASD group and controls were not statistically significant. These results suggest that individuals with ASD are more likely to have a higher proportion of body fat during childhood and pre-adolescence, indicating distinct differences in body composition between the two groups. Detailed results from the bioelectrical impedance analysis performed on the participants are included in Table 4.

### 3.4. Determinants of Overweight/Obesity in ASD Group

Univariate analysis showed that gender, age, and geographic location were not potential risk factors associated with the occurrence of obesity/overweight, as shown in Table 5 (*p* > 0.05).

Multivariate analysis (Table 6) demonstrated that none of the predictor variables (gender, age, and geographic location) showed a statistically significant effect on the likelihood of being obese/overweight.

## 4. Discussion

The purpose of this study was to examine the differences in anthropometric measures and body composition variables of Lebanese ASD children and adolescents compared to their neurotypical counterparts across various developmental stages. It also investigated the occurrence of unhealthy weight and identified associated risk factors within the ASD population.

Our findings revealed a notable imbalance in gender distribution, with males comprising a larger proportion compared to females (86% vs. 14%, respectively). This observation aligns with prior research indicating a higher prevalence of ASD among males than females [41,42]. This disparity in prevalence rates highlights a potential gender bias in the typical presentation of ASD [43]. This bias may stem from the original development and testing of diagnostic tools primarily with male participants, which could contribute to misdiagnosis in females [44]. Consequently, females with ASD might need to manifest a higher number of or more severe symptoms to be diagnosed. Moreover, females with ASD often engage in “camouflaging” their autistic symptoms more than males, making it harder to detect their condition [45]. As a result, many females may be misdiagnosed, experience significant delays in diagnosis, or go undiagnosed altogether, leading to a lack of necessary intervention and support.

Additionally, we found that individuals with ASD were at a higher risk of developing unhealthy weight compared to their neurotypical counterparts. The occurrence rates of overweight and obesity among this population were alarming, prompting urgent attention to address this growing clinical concern. In fact, our study revealed that 19.8% of ASD participants were classified as overweight, whereas 34.9% were classified as obese. Taken together, this high prevalence emphasizes the critical need for targeted interventions designed to address weight-related health challenges. Interestingly, our findings align with previous studies on the prevalence of overweight and obesity in ASD (Table 7).

Memari et al. (2012) conducted a study focusing on the weight status of Iranian children and adolescents diagnosed with ASD. Their research revealed that 23.1% of the population were classified as overweight, while 24.1% were categorized as obese [46]. Similarly, Bicer and Alsaffar et al. (2013) noted an increased susceptibility to overweight and obesity among adolescents with ASD [47]. Consistent findings were observed by Broder-Fingert et al., indicating that children with ASD exhibited higher rates of overweight and obesity compared to control subjects [48]. Additionally, Zuckerman et al. assessed the prevalence of overweight and obesity among 376 ASD children, revealing that. 18.1% were classified as overweight and 17% as obese [49]. Moreover, Healy et al. highlighted that 19.4% were overweight and 23.05 were obese [51]. The underlying reasons for the high prevalence of overweight and obesity in children with ASD are still not fully understood. Previous studies have highlighted that the increased prevalence of unhealthy weight among children with ASD is often linked to atypical eating patterns [52]. In fact, children with ASD, often referred to as picky eaters, experience more feeding difficulties compared to typically developing controls [53]. They tend to eat selectively, demonstrating a preference for starches, fast food, sugary beverages, and snacks over fruits and vegetables [54]. This dietary preference can predispose them to an imbalanced body composition. Furthermore, reduced physical activity and highly sedentary behavior are key contributors to weight gain and increased BMI [26]. In fact, children diagnosed with ASD commonly experience motor impairments, which can hinder their engagement in sports and physical activities. These impairments include diminished muscle tone, oral motor challenges, instability in posture, and difficulties in motor skills [52]. Moreover, challenges in social skills often result in reduced participation in structured activities with peers, further limiting their physical activity [55]. Additionally, the use of antipsychotic medications, often prescribed for managing ASD symptoms, presents a risk factor for weight gain in this population. The medication linked to the most pronounced weight gain is olanzapine, followed with clozapine, risperidone, and aripiprazole, in ascending order of weight gain severity [56] (Table 8).

Genetic factors implicated in obesity, such as 11p14.1 or 16p11.2 microdeletions, may also be prevalent among children diagnosed with ASD [62]. It is crucial to acknowledge that elevated BMI levels are correlated with adverse health consequences, such as insulin resistance, heart disease, diabetes, and sleep-disordered breathing [63]. Childhood obesity imposes a significant economic burden on families and can have detrimental effects on physical, emotional, and social well-being, as well as academic performance, potentially exacerbating the challenges posed by ASD-related disability and leading to a diminished quality of life [26]. In fact, overweight or obese children with ASD are more prone to experience bullying and social isolation [27]. These findings underscore the imperative need for early intervention and comprehensive support tailored to the unique challenges related to weight and overall health in individuals with ASD.

When analyzing the prevalence of obesity across various age brackets, our study revealed a higher incidence of overweight and obesity among young children aged 3 to 9 years and pre-adolescents aged 10 to 13 years diagnosed with ASD compared to their neurotypical counterparts. Additionally, we found no significant difference in obesity prevalence between adolescents aged 14 to 18 years with ASD and their typically developing peers. These findings contrast with some previous studies. For example, Tybor et al. (2019) noted a significantly higher obesity prevalence among ASD children aged 10 to 17 years compared to those without ASD [64]. Similarly, McCoy et al. (2019) reported that ASD adolescents aged 10 to 17 years were more likely to exhibit unhealthy weight statuses, including underweight, overweight, and obesity, compared to their typically developing counterparts [65]. Moreover, Evans et al. (2012) investigated the body mass index of ASD and control individuals aged 3 to 11 years and found no discernible differences in BMI z-scores or BMI cut-points between the two groups [66]. Furthermore, Barnhill et al. (2017) studied the weight status of ASD children aged 2 to 13 years and reported no significant disparities in BMI categories compared to age-matched controls [67]. These inconsistencies, particularly observed in adolescence, underscore the necessity for further research to explore the impact of puberty on obesity in individuals with ASD. Understanding how developmental changes influence weight status in this population is crucial for developing tailored interventions.

Furthermore, bioelectrical impedance analysis (BIA) revealed variations in body composition between the two groups across different developmental stages. During childhood and pre-adolescence, individuals with ASD exhibited significantly higher levels of fat mass (FM) and total body fat percentage (PBF) compared controls. Interestingly, these differences were not observed during adolescence. Similarly, the levels of fat-free mass (FFM) showed no significant difference across all age groups. These findings suggest age-related variations in body composition. In fact, younger children and pre-adolescents diagnosed with ASD exhibited a greater proportion of body fat, indicating overweight, necessitating targeted interventions to address dietary habits, physical activity levels, and sensory sensitivities that may influence these outcomes. Throughout adolescence, both individuals with ASD and typically developing controls experience substantial rapid physical growth and hormonal fluctuations [68]. It may be possible that these changes affect both groups similarly, thereby reducing the observed differences in body composition and anthropometric measurements.

Moreover, we investigated the relationship between sociodemographic characteristics and the occurrence of overweight and obesity in ASD children. Our analysis indicated that variables such as age, gender, and geographic location did not exhibit significant associations with weight status among this population. This finding is consistent with previous studies. In fact, Healy et al. did not identify any correlation between age and the occurrence of obesity in children diagnosed with ASD. Similarly, Sammels et al. found no relationship between the prevalence of obesity and the gender of the children with ASD [69]. Future research should investigate the association between the occurrence of obesity and several factors, including the severity of ASD diagnosis, medication use, as well as levels of physical activity, dietary habits, and sleep patterns.

We acknowledge several limitations in this study. The first is the relatively smaller sample size of adolescents compared to younger children and pre-adolescents. Factors like medication use, physical activity levels, which could have provided valuable insights, and dietary intake were not evaluated. Additionally, the measurement parameters of BIA are susceptible to variations caused by factors such as physical activity, fluid and food intake, hydration status, and electrode placement methods, potentially leading to inaccuracies in BIA readings [70]. However, to ensure the accuracy and reliability of our BIA measurements, we implemented stringent protocols. Before testing, participants were advised to fast overnight, empty their bladders, and avoid exercise for 24 h. Therefore, future research should aim to incorporate validated methods for assessing pubertal status, such as Tanner staging or hormone level measurements, to better understand its impact on weight-related outcomes in this population. Moreover, future research could focus on examining the correlation between medication use, physical activity levels, dietary intake, and the prevalence of obesity and overweight in ASD individuals.

## 5. Conclusions

In conclusion, our study has shown that individuals with ASD exhibit alarming rates of overweight and obesity during childhood and pre-adolescence when compared to their neurotypical counterparts. This finding highlights the urgent need for targeted interventions to address weight-related health challenges in this population. Furthermore, distinct disparities in body composition during childhood and pre-adolescence were evident between the two groups. However, no differences in FM, FFM, and PBF were observed in adolescents between the groups. Future research could focus on evaluating factors such as medication use, physical activity, and dietary intake. Additionally, incorporating validated methods for assessing pubertal status, such as Tanner staging or hormone level measurements, could provide deeper insights into its influence on weight-related outcomes in this population. These investigations hold promise for enhancing their quality of life and overall well-being.

## Figures and Tables

**Table 1:** Intervals of the z-scores.

Classification	Z-Score Values
Weight-for-age z-score (WAZ)	
Low weight	Z < −2 SD
Normal weight	−2 SD < Z < +2 SD
Overweight	Z > +2 SD
Height-for-age z-score (WAZ)	
Stunted	Z < −2 SD
Normal height	−2 SD < Z < +2 SD
Too tall	Z > +2 SD
BMI-for-age z-score (BMIZ)	
Underweight	Z < −1.64 SD
Normal weight	−1.64 SD < Z < +1 SD
Overweight	+1 SD < Z < +1.64 SD
Obese	Z > +1.64 SD

**Table 2:** Sociodemographic data of the study and control groups.

Variables	ASD Group (*n* = 86)	Control Group (*n* = 86)
Age (year) *	10.4 ± 3.51	10.4 ± 3.51
Age (months) *	128.94 ± 42.08	128.96 ± 42.36
Age category **		
Young children (3–9 years old)	35 (40.7%)	35 (40.7%)
Pre-adolescents (10–13 years old)	36 (41.9%)	36 (41.9%)
Adolescents (14–18 years old)	15 (17.4%)	15 (17.4%)
Gender **		
Male	74 (86%)	74 (86%)
Female	12 (14%)	12 (14%)
Geographic location **		
Beirut	18 (20.9%)	18 (20.9%)
Bekaa	19 (22.1%)	19 (22.1%)
North	31 (36.1%)	31 (36.1%)
South	18 (20.9%)	18 (20.9%)

* Continuous variable is presented as mean ± standard deviation. ** Categorical variables are summarized as frequencies (*n*) and percentage (%).

**Table 3:** Anthropometric measurements stratified according to different age categories in both groups.

Variables	Young Children (*n* = 70)		*p*-Value	Pre-Adolescents (*n* = 72)		*p*-Value	Adolescents (*n* = 30)		*p*-Value
	ASD (*n* = 35)	Control (*n* = 35)		ASD (*n* = 36)	Control (*n* = 36)		ASD (*n* = 15)	Control (*n* = 15)	
Weight (kg) ^2^	29.7 ± 8.72	24.45 ± 7.54	0.008 *	50.88 ± 16.51	39.57 ± 12.66	0.000 *	70.44 ± 22.74	67.15 ± 22	0.740
WAZ ^1^	0.97 ± 1.05	−0.24 ± 1.22	0.000 *	0.83 ± 1.09	−0.48 ± 1.38	0.000 *	0.66 ± 1.55	0.45 ± 1.31	0.691
WAZ cut points ^2^			0.010 *			0.004 *			0.595
Low weight	0 (0%)	2 (6%)		0 (0%)	7 (19%)		1 (7%)	0 (0%)	
Normal weight	28 (80%)	32 (91%)		32 (89%)	28 (78%)		12 (80%)	12 (80%)	
Overweight	7 (20%)	1 (3%)		4 (11%)	1 (3%)		2 (13%)	3 (20%)	
Height (cm) ^1^	124.43 ± 10.38	122.76 ± 12.84	0.550	149.49 ± 14.13	146.33 ± 9.32	0.267	165.08 ± 1.56	169.02 ± 11.73	0.342
HAZ ^2^	0.27 ± 1.32	−0.55 ± 1.34	0.431	0.02 ± 1.63	−0.47 ± 1.18	0.031 *	−0.56 ± 1.17	−0.06 ± 1.3	0.520
HAZ cut-points ^2^			0.322			0.746			0.087
Stunted	1 (3%)	3 (3%)		3 (8%)	2 (6%)		2 (13%)	0 (0%)	
Normal height	31 (88%)	30 (86%)		30 (84%)	31 (86%)		13 (87%)	14 (93%)	
Too tall	3 (9%)	2 (1%)		3 (8%)	3 (8%)		0 (0%)	1 (7%)	
BMI (kg/m^2^) ^2^	18.8 ± 3.73	15.9 ± 2.78	0.000 *	22.45 ± 4.68	18.19 ± 4.41	0.000 *	25.5 ± 6.98	23.35 ± 6.71	0.389
BMIZ ^1^	1.05 ± 0.92	−0.31 ± 1.25	0.000 *	1.04 ± 1.02	−0.37 ± 1.39	0.000 *	0.8 ± 1.45	0.32 ± 1.38	0.355
BMIZ cut points ^2^			0.000 *			0.000 *			0.345
Underweight	0 (0%)	3 (9%)		1 (3%)	8 (22%)		1 (6.7%)	2 (13%)	
Healthy weight	17 (49%)	29 (83%)		14 (39%)	21 (58%)		6 (40%)	6 (40%)	
Overweight	8 (22%)	1 (2%)		8 (22%)	4 (11%)		1 (6.7%)	4 (27%)	
Obese	10 (29%)	2 (6%)		13 (36%)	3 (9%)		7 (46.6%)	3 (20%)	
Overweight/obese	18 (51%)	3 (8%)		21 (58%)	7 (20%)		8 (53.3)	7 (47%)	
WC (cm)^2^	67.14 ± 12.46	58.23 ± 7.83	0.000 *	79.86 ± 13.48	70.86 ± 13.77	0.006 *	90.2 ± 18.43	84.76 ± 17.37	0.345

Continuous variables are expressed as mean ± standard deviation (SD), while categorical variables are presented as frequencies (*n*) and percentages (%). Z-scores were calculated based on reference values for the same sex and age. The abbreviations used are as follows: WAZ for weight-for-age z-score, HAZ for height-for-age z-score, BMIZ for BMI-for-age z-score, and WC for waist circumference. ^1^ The independent *t*-test was utilized to compare variables that are normally distributed. ^2^ The Mann–Whitney test was used for variables with non-normal distributions. * A *p*-value of less than 0.05 was considered statistically significant.

**Table 4:** Bioelectrical impedance analysis of the participants.

Variables	Young Children (*n* = 70)		*p*-Value	Pre-Adolescents (*n* = 72)		*p*-Value	Adolescents (*n* = 30)		*p*-Value
	ASD (*n* = 35)	Control (*n* = 35)		ASD (*n* = 36)	Control (*n* = 36)		ASD (*n* = 35)	Control (*n* = 35)	
FM (kg)	3.89 ± 3.49	1.74 ± 2.35	0.000 *	9.31 ±6.18	4.18 ± 5.24	0.000 *	14.63 ± 12.71	10.27 ± 8.70	0.486
FFM (kg)	25.77 ± 6.04	23.85 ± 8.83	0.104	41.55 ± 12.37	35.39 ± 9.43	0.000 *	55.81 ± 13.07	55.75 ± 17.56	0.902
PBF (%)	11.66 ± 6.75	6.28 ± 5.2	0.000 *	17.26 ± 9.02	8.91 ± 7.72	0.000 *	17.94 ± 13.26	12.81 ± 9.24	0.345

Continuous variables are presented as mean ± standard deviation with statistical comparison using *t*-test. * Significantly different at *p*-value < 0.05. FM: fat mass; FFM: fat-free mass; PBF: percent body fat.

**Table 5:** Univariate analysis for determinants of obesity in the ASD group.

Risk Factor	Pearson Chi-Square Value	*p*-Value
Gender	0.812	0.367
Age category	0.892	0.640
Geographic location	4.188	0.242

Significantly different at *p*-value < 0.05.

**Table 6:** Multivariate analysis of risk factors predicting obesity status in ASD group.

Risk Factor	Odds Ratio	95% Confidence Interval	*p*-Value
Gender	0.538	0.147–1.965	0.348
Age category	1.322	0.729–2.397	0.359
Geographic location	0.899	0.593–1.361	0.614

Significantly different at *p*-value < 0.05.

**Table 7:** Overview of the prevalence of overweight and obesity in ASD.

Source	Location	Age Range	Sample Size	Weight/Height Measures	Overweight (%)	Obese (%)
(Memari et al., 2012) [46]	Iran	7–14	113	Measured	23.1	24.1
(Bicer and Alsaffar, 2013) [47]	Turkey	4–18	164	Measured	26.2	32.3
(Broder-Fingert et al., 2014) [48]	Eastern Massachusetts	2–20	2075	Measured	14.8	23.2
(Zuckerman et al., 2014) [49]	United States and Canada	2–17	376	Measured	18.1	17
(Curtin et al., 2015) [50]	-	3–17	85,272	Parent-reported	-	23.6
(Healy et al., 2019) [23]	United States	10–17	875,963	Parent-reported	19.4	23.05
(Kamal Nor et al., 2019) [51]	Malaysia	2–18	151	Measured	11.3	21.9

**Table 8:** Summary of the side effects of antipsychotic drug treatments in children with ASD.

Source	Age Range	Sample Size	Treatment	Duration	Results
(Malone et al., 2001) [57]	5–17 years old	12	Olanzapine and haloperidol	6 weeks	From baseline to the end of treatment: the olanzapine group demonstrated greater weight gain compared to the haloperidol group
(Yoon et al., 2016) [58]	2–20 years old	202	One of five (Olanzapine, aripiprazole, risperidone, quetiapine, and ziprasidone)	Up to 4 years	Olanzapine, aripiprazole, and risperidone all led to an increase in BMI z-score
(Scahill et al., 2016) [59]	4–13 years old	124	Risperidone	24 weeks	Weight gain and increased waist circumference were reported in patients treated with risperidone
(Ceylan et al., 2020) [60]	-	102	One of three (olanzapine, risperidone, and aripiprazole)	8 weeks	Weight gain occurred with all three antipsychotics, with olanzapine demonstrating the highest frequency compared to the others
(Vanwong et al., 2020) [61]	3–18 years old	134	Risperidone	3 months and more	Children and adolescents with ASD treated with risperidone exhibited significantly higher rates of obesity and overweight than their healthy individuals

## Data Availability

Data are contained within the article.

## References

[1] APA (2013). Diagnostic and Statistical Manual of Mental Disorders.

[2] Nag H.E., Nordgren A., Anderlid B.-M., Nærland T. (2018). Reversed Gender Ratio of Autism Spectrum Disorder in Smith-Magenis Syndrome. Mol. Autism.

[3] Salari N., Rasoulpoor S., Rasoulpoor S., Shohaimi S., Jafarpour S., Abdoli N., Khaledi B., Mohammadi M. (2022). The Global Prevalence of Autism Spectrum Disorder: A Comprehensive Systematic Review and Meta-Analysis. Riv. Ital. Di Pediatr. Ital. J. Pediatr..

[4] Zeidan J., Fombonne E., Scorah J., Ibrahim A., Durkin M.S., Saxena S., Yusuf A., Shih A., Elsabbagh M. (2022). Global Prevalence of Autism: A Systematic Review Update. Autism Res..

[5] Richa S., Khoury R., JRouhayem J., Chammay R., Kazour F., Khalil R.B., Kheir W., Choueifaty D., Kouba-Hreich E., Gerbaka B. (2020). Estimating the Prevalence of Autism Spectrum Disorder in Lebanon. L’Encéphale.

[6] Bitar T., Hleihel W., Marouillat S., Vonwill S., Vuillaume M., Soufia M., Vourc’h P., Laumonnier F., Andres C.R. (2019). Identification of Rare Copy Number Variations Reveals PJA2, APCS, SYNPO, and TAC1 as Novel Candidate Genes in Autism Spectrum Disorders. Mol. Genet. Genom. Med..

[7] Gerges P., Bitar T., Hawat M., Alameddine A., Soufia M., Andres C.R., Hleihel W. (2020). Risk and Protective Factors in Autism Spectrum Disorders: A Case Control Study in the Lebanese Population. Int. J. Environ. Res. Public Health.

[8] Ivanov H., Stoyanova V., Popov N., Vachev T. (2015). Autism Spectrum Disorder—A Complex Genetic Disorder. Folia Medica.

[9] Lyall K., Croen L., Daniels J., Fallin M.D., Ladd-Acosta C., Lee B.K., Park B.Y., Snyder N.W., Schendel D., Volk H. (2017). The Changing Epidemiology of Autism Spectrum Disorders. Annu. Rev. Public Health.

[10] Khachadourian V., Mahjani B., Sandin S., Kolevzon A., Buxbaum J., Reichenberg A., Janecka M. (2023). Comorbidities in Autism Spectrum Disorder and Their Etiologies. Transl. Psychiatry.

[11] Chistol L.T., Bandini L.G., Must A., Phillips S., Cermak S.A., Curtin C. (2018). Sensory Sensitivity and Food Selectivity in Children with Autism Spectrum Disorder. J. Autism Dev. Disord..

[12] Na C., Watanabe K., Kobayakawa T., Wada M. (2022). Relationships between Autistic Traits, Taste Preference, Taste Perception, and Eating Behaviour. Eur. Eat. Disord. Rev. J. Eat. Disord. Assoc..

[13] Zulkifli M.N., Kadar M., Fenech M., Hamzaid N.H. (2022). Interrelation of Food Selectivity, Oral Sensory Sensitivity, and Nutrient Intake in Children with Autism Spectrum Disorder: A Scoping Review. Res. Autism Spectr. Disord..

[14] Dijk M., Buruma M., Blijd-Hoogewys E. (2021). Detecting Feeding Problems in Young Children with Autism Spectrum Disorder. J. Autism Dev. Disord..

[15] Baraskewich J., von Ranson K.M., McCrimmon A., McMorris C.A. (2021). Feeding and Eating Problems in Children and Adolescents with Autism: A Scoping Review. Autism.

[16] Crasta J., Benjamin T., Catherine A., Jemi M., Kanniappan G., Padankatti S., Russell P., Nair M. (2014). Feeding Problems Among Children with Autism in a Clinical Population in India. Indian J. Pediatr..

[17] Fildes A., Mallan K.M., Cooke L., Van Jaarsveld C.H., Llewellyn C.H., Fisher A., Daniels L. (2015). The Relationship between Appetite and Food Preferences in British and Australian Children. Int. J. Behav. Nutr. Phys. Act..

[18] Park H.J., Choi S.J., Kim Y., Cho M.S., Kim Y.-R., Oh J.E. (2021). Mealtime Behaviors and Food Preferences of Students with Autism Spectrum Disorder. Foods.

[19] Esposito M., Mirizzi P., Fadda R., Pirollo C., Ricciardi O., Mazza M., Valenti M. (2023). Food Selectivity in Children with Autism: Guidelines for Assessment and Clinical Interventions. Int. J. Environ. Res. Public Health.

[20] Alkhalidy H., Abushaikha A., Alnaser K., Obeidat M.D., Al-Shami I. (2021). Nutritional Status of Pre-School Children and Determinant Factors of Autism: A Case-Control Study. Front. Nutr..

[21] Castro K., Faccioli L.S., Baronio D., Gottfried C., Perry I.S., Riesgo R. (2017). Body Composition of Patients with Autism Spectrum Disorder through Bioelectrical Impedance. Nutr. Hosp..

[22] Chen A., Kim S., Houtrow A., Newacheck P. (2009). Prevalence of Obesity Among Children with Chronic Conditions. Obesity.

[23] Healy S., Aigner C., Haegele J. (2018). Prevalence of Overweight and Obesity among US Youth with Autism Spectrum Disorder. Autism.

[24] Sedgewick F., Leppanen J., Tchanturia K. (2020). Autistic Adult Outcomes on Weight and Body Mass Index: A Large-Scale Online Study. Eat. Weight Disord. Stud. Anorex. Bulim. Obes..

[25] Curtin C., Jojic M., Bandini L.G. (2014). Obesity in Children with Autism Spectrum Disorders. Harv. Rev. Psychiatry.

[26] Dhaliwal K.K., Orsso C.E., Richard C., Haqq A.M., Zwaigenbaum L. (2019). Risk Factors for Unhealthy Weight Gain and Obesity among Children with Autism Spectrum Disorder. Int. J. Mol. Sci..

[27] Hill A.P., Zuckerman K.E., Fombonne E. (2015). Obesity and Autism. Pediatrics.

[28] World Health Organization (2018). Noncommunicable Diseases Country Profiles 2018.

[29] Başıbüyük G.Ö., Ayremlou P., Saeidlou S.N., Ay F., Dalkıran A., Simzari W., Vitályos G.Á., Bektaş Y. (2021). A Comparison of the Different Anthropometric Indices for Assessing Malnutrition among Older People in Turkey: A Large Population-Based Screening. J. Health Popul. Nutr..

[30] Orgel E., Mueske N.M., Sposto R., Gilsanz V., Freyer D.R., Mittelman S.D. (2018). Limitations of Body Mass Index to Assess Body Composition Due to Sarcopenic Obesity during Leukemia Therapy. Leuk. Lymphoma.

[31] Duren D., Sherwood R., Czerwinski S., Lee M., Choh A., Siervogel R., Chumlea W. (2008). Body Composition Methods: Comparisons and Interpretation. J. Diabetes Sci. Technol..

[32] Xu H., Liu J., Zhang X., Xue Y., Shi J., Chen W., Zheng X. (2021). Estimation of Skeletal Muscle Mass by Bioimpedance and Differences among Skeletal Muscle Mass Indices for Assessing Sarcopenia. Clin. Nutr..

[33] Ryu H., Park H.C., Kim H., Heo J., Kang E., Hwang Y.-H., Cho J.Y., Lee K.-B., Oh Y.K., Oh K.-H. (2019). Bioelectrical Impedance Analysis as a Nutritional Assessment Tool in Autosomal Dominant Polycystic Kidney Disease. PLoS ONE.

[34] Breehl L., Caban O. (2023). Physiology, Puberty. StatPearls.

[35] Esteban-Figuerola P., Morales-Hidalgo P., Arija-Val V., Canals-Sans J. (2021). Are There Anthropometric and Body Composition Differences between Children with Autism Spectrum Disorder and Children with Typical Development? Analysis by Age and Spectrum Severity in a School Population. Autism.

[36] Salehi1 H., Aghanoori M.R., Shahmohammadlu S., Hosseini B., Mitchell S., Mahmudi M., Djafarian K. (2015). Body Composition in Iranian Boys with Autism Spectrum Disorders. Paediatr. Croat..

[37] Trambacz S. (2021). Weight Status and Body Composition Analysis among Polish Boys with Autism Spectrum Disorders. Anthropol. Rev..

[38] Chou J., Roumiantsev S., Singh R. (2019). PediTools LMS-Based Anthropometric Calculators: Applications in Clinical Care, Research, and Quality Improvement (Preprint). J. Med. Internet Res..

[39] Akın Koç S., GARİPAĞAOĞLU M., EKİNCİ Ö., KANIK A., Gültekin F. (2023). Nutritional and Obesity Status of Children and Adolescents with ADHD: A Case-Control Study. Bangladesh J. Med. Sci..

[40] Premkumar S., Venkatramanan P., Dhivyalakshmi J., Thiruvengadam G. (2019). Comparison of Nutrition Status as Assessed by Revised IAP 2015 Growth Charts and CDC 2000 Growth Charts in Lower Socioeconomic Class School Children. Indian J. Pediatr..

[41] de Giambattista C., Ventura P., Trerotoli P., Margari F., Margari L. (2021). Sex Differences in Autism Spectrum Disorder: Focus on High Functioning Children and Adolescents. Front. Psychiatry.

[42] Schuck R.K., Flores R.E., Fung L.K. (2019). Brief Report: Sex/Gender Differences in Symptomology and Camouflaging in Adults with Autism Spectrum Disorder. J. Autism. Dev. Disord..

[43] Kirkovski M., Enticott P.G., Fitzgerald P.B. (2013). A Review of the Role of Female Gender in Autism Spectrum Disorders. J. Autism. Dev. Disord..

[44] Kreiser N.L., White S.W. (2014). ASD in Females: Are We Overstating the Gender Difference in Diagnosis?. Clin. Child Fam. Psychol. Rev..

[45] Hull L., Petrides K.V., Allison C., Smith P., Baron-Cohen S., Lai M.-C., Mandy W. (2017). “Putting on My Best Normal”: Social Camouflaging in Adults with Autism Spectrum Conditions. J. Autism Dev. Disord..

[46] Memari A.H., Kordi R., Ziaee V., Mirfazeli F.S., Setoodeh M.S. (2012). Weight Status in Iranian Children with Autism Spectrum Disorders: Investigation of Underweight, Overweight and Obesity. Res. Autism Spectr. Disord..

[47] Bicer A.H., Alsaffar A.A. (2013). Body Mass Index, Dietary Intake and Feeding Problems of Turkish Children with Autism Spectrum Disorder (ASD). Res. Dev. Disabil..

[48] Broder-Fingert S., Brazauskas K., Lindgren K., Iannuzzi D., Van Cleave J. (2014). Prevalence of Overweight and Obesity in a Large Clinical Sample of Children with Autism. Acad. Pediatr..

[49] Zuckerman K.E., Hill A.P., Guion K., Voltolina L., Fombonne E. (2014). Overweight and Obesity: Prevalence and Correlates in a Large Clinical Sample of Children with Autism Spectrum Disorder. J. Autism. Dev. Disord..

[50] Curtin C., Hubbard K., Anderson S.E., Mick E., Must A., Bandini L.G. (2015). Food Selectivity, Mealtime Behavior Problems, Spousal Stress, and Family Food Choices in Children with and without Autism Spectrum Disorder. J. Autism. Dev. Disord..

[51] Kamal Nor N., Ghozali A.H., Ismail J. (2019). Prevalence of Overweight and Obesity Among Children and Adolescents with Autism Spectrum Disorder and Associated Risk Factors. Front. Pediatr..

[52] Curtin C., Anderson S.E., Must A., Bandini L. (2010). The Prevalence of Obesity in Children with Autism: A Secondary Data Analysis Using Nationally Representative Data from the National Survey of Children’s Health. BMC Pediatr..

[53] Adams S.N. (2022). Feeding and Swallowing Issues in Autism Spectrum Disorders. Neuropsychiatr. Dis. Treat..

[54] Zulkifli M.N., Kadar M., Hamzaid N.H. (2022). Weight Status and Associated Risk Factors of Mealtime Behaviours among Children with Autism Spectrum Disorder. Children.

[55] Memari A.H., Mirfazeli F.S., Kordi R., Shayestehfar M., Moshayedi P., Mansournia M.A. (2017). Cognitive and Social Functioning Are Connected to Physical Activity Behavior in Children with Autism Spectrum Disorder. Res. Autism Spectr. Disord..

[56] Cuda S., Censani M., Kharofa R., O’Hara V., Conroy R., Williams D.R., Paisley J., Browne A.F., Karjoo S., Browne N.T. (2022). Medication-Induced Weight Gain and Advanced Therapies for the Child with Overweight and Obesity: An Obesity Medicine Association (OMA) Clinical Practice Statement 2022. Obes. Pillars.

[57] Malone R.P., Cater J., Sheikh R.M., Choudhury M.S., Delaney M.A. (2001). Olanzapine Versus Haloperidol in Children with Autistic Disorder: An Open Pilot Study. J. Am. Acad. Child Adolesc. Psychiatry.

[58] Yoon Y., Wink L.K., Pedapati E.V., Horn P.S., Erickson C.A. (2016). Weight Gain Effects of Second-Generation Antipsychotic Treatment in Autism Spectrum Disorder. J. Child. Adolesc. Psychopharmacol..

[59] Scahill L., Jeon S., Boorin S., McDougle C., Aman M., Dziura J., McCracken J., Caprio S., Arnold L.E., Nicol G. (2016). Weight Gain and Metabolic Consequences of Risperidone in Young Children with Autism Spectrum Disorder. J. Am. Acad. Child Adolesc. Psychiatry.

[60] Tural Hesapçıoğlu S., Ceylan M., Kaşak M., Yavaş C. (2020). Olanzapine, Risperidone, and Aripiprazole Use in Children and Adolescents with Autism Spectrum Disorders. Res. Autism Spectr. Disord..

[61] Vanwong N., Ngamsamut N., Nuntamool N., Hongkaew Y., Sukprasong R., Puangpetch A., Limsila P., Sukasem C. (2020). Risperidone-Induced Obesity in Children and Adolescents with Autism Spectrum Disorder: Genetic and Clinical Risk Factors. Front. Pharmacol..

[62] Ranieri A., Mennitti C., Falcone N., La Monica I., Di Iorio M.R., Tripodi L., Gentile A., Vitale M., Pero R., Pastore L. (2023). Positive Effects of Physical Activity in Autism Spectrum Disorder: How Influences Behavior, Metabolic Disorder and Gut Microbiota. Front. Psychiatry.

[63] Leinum C.J., Dopp J.M., Morgan B.J. (2009). Sleep Disordered Breathing and Obesity: Pathophysiology, Complications and Treatment. Nutr. Clin. Pract..

[64] Tybor D.J., Eliasziw M., Kral TV E., Segal M., Sherwood N.E., Sikich L., Stanish H., Bandini L., Curtin C., Must A. (2019). Parental concern regarding obesity in children with autism spectrum disorder in the United States: National Survey of Children’s Health 2016. Disabil. Health J..

[65] McCoy S.M., Morgan K. (2020). Obesity, physical activity, and sedentary behaviors in adolescents with autism spectrum disorder compared with typically developing peers. Autism.

[66] Evans E.W., Must A., Anderson S.E., Curtin C., Scampini R., Maslin M., Bandini L. (2012). Dietary patterns and body mass index in children with autism and typically developing children. Res. Autism Spectr. Disord..

[67] Barnhill K., Gutierrez A., Ghossainy M., Marediya Z., Marti C.N., Hewitson L. (2017). Growth status of children with autism spectrum disorder: A case–control study. J. Hum. Nutr. Diet..

[68] Siervogel RM Demerath E.W., Schubert C., Remsberg K.E., Chumlea W.C., Sun S., Czerwinski S.A., Towne B. (2004). Puberty and Body Composition. Horm. Res..

[69] Sammels O., Karjalainen L., Dahlgren J., Wentz E. (2022). Autism Spectrum Disorder and Obesity in Children: A Systematic Review and Meta-Analysis. Obes. Facts.

[70] Wasyluk W., Wasyluk M., Zwolak A., Łuczyk R.J. (2019). Limits of body composition assessment by bioelectrical impedance analysis (BIA). J. Educ. Health Sport.

